# Community-engaged and Implementation Science-informed Pediatric Sleep Intervention Research

**DOI:** 10.1007/s40675-026-00389-5

**Published:** 2026-07-13

**Authors:** Ariel A. Williamson, Jessica C. Levenson

**Affiliations:** 1 The Ballmer Institute for Children’s Behavioral Health, University of Oregon, 2800 NE Liberty Street, Portland, OR 97211 USA; 2 Department of Psychology, College of Arts and Sciences, University of Oregon, Eugene, OR USA; 3 Department of Pediatrics, Institute on Development and Disabilities, Oregon Health & Science University, Portland, OR USA; 4 Department of Psychiatry, University of Pittsburgh School of Medicine, Pittsburgh, PA USA; 5 Department of Pediatrics, University of Pittsburgh School of Medicine, Pittsburgh, PA USA; 6 Clinical and Translational Science Institute, University of Pittsburgh, Pittsburgh, PA USA

**Keywords:** Adolescent, Child, Community-engaged research, Implementation science, Intervention, Sleep

## Abstract

**Purpose of Review:**

Community-engaged research (CEnR) and implementation science can address gaps in translating evidence-based interventions to real-world practice by considering the needs of patients/families, clinicians, and contexts. This review summarizes recent pediatric sleep intervention research that incorporates CEnR and/or implementation science principles. We also identify key areas for future research.

**Recent Findings:**

Most reviewed studies incorporated CEnR methods during intervention development, adaptation, and/or pilot testing. Studies with community members collaborating in named research team roles were rare. Although some studies evaluated intervention acceptability, feasibility, and fidelity, very few used established implementation science theories, models, or frameworks, and even fewer focused on developing and testing implementation strategies.

**Summary:**

It is critical to establish sleep researcher-community partnerships that promote community empowerment and capacity-building, and to conduct implementation strategy research. We encourage researchers to further integrate CEnR and implementation science to advance pediatric sleep health and address the many gaps in care.

## Introduction

Pediatric sleep problems, including insomnia symptoms (difficulty falling/staying asleep), sleep disordered breathing (SDB; obstructive sleep apnea), and poor sleep health (insufficient and/or irregular duration) [1], are highly prevalent from early childhood through adolescence [2–5]. Sleep problems occur in 15–40% of children and adolescents across ages [2, 4–6] and tend to persist without treatment [7]. Fortunately, cognitive and behavioral strategies can address insomnia and poor sleep health [5, 8–11], while adenotonsillectomy [12] and/or positive airway pressure [13] can effectively treat pediatric obstructive sleep apnea. Early identification and treatment of sleep problems is especially important given that untreated sleep problems are linked to adverse neurobehavioral, academic, and mental health outcomes [14–16].

Despite evidence of effective pediatric sleep interventions, there are significant gaps in identifying and treating sleep problems. Electronic health record (EHR) [6, 17, 18] and insurance claims [19–21] data show incredibly low rates of sleep disorder diagnosis in primary care and outpatient practice more broadly. Whereas conservative estimates suggest at least 10% of children have diagnosable insomnia [2], EHR and claims data show only 2–4% of children have any sleep disorder diagnosis [17, 19]. Even when sleep problems are diagnosed, few children receive evidence-based treatment [21, 22]. Furthermore, there are racial, ethnic, and socioeconomic sleep health disparities across development [23]. For example, compared to their White peers, African American/Black and Hispanic/Latine youth are more likely to experience insufficient sleep [24] and sleep disordered breathing [25], yet less likely to receive treatment [26]. Those living in lower socioeconomic status (SES) homes and neighborhoods and/or in rural contexts experience similar sleep health disparities compared to children in higher income and/or large, metropolitan contexts [27–29].

Several factors contribute to gaps in the under-identification and limited evidence-based treatment of pediatric sleep problems. Overall, there is a shortage of medical and behavioral clinicians with pediatric sleep training [30], with very limited training opportunities available during graduate and medical education [31–33]. Additionally, few studies have examined optimal methods to train non-specialty sleep clinicians in existing evidence-based sleep practices, limiting dissemination [34]. There are also few studies of intervention implementation and effectiveness with populations experiencing sleep health disparities [23, 35], and in non-research settings (i.e., outpatient clinics with varying levels of resources and clinician expertise) [34]. Qualitative research suggests that adaptations to behavioral sleep interventions may be needed to enhance acceptability and outcomes in health disparity populations [36–38], due to cultural and contextual factors [23]. Adaptations may also be needed for youth experiencing co-occurring sleep problems and medical, neurodevelopmental, and/or mental health conditions [39, 40].

Developing and testing pediatric sleep interventions without considering the needs of heterogeneous patient and family populations (i.e., end recipients), clinicians (i.e., end users), and contexts (i.e., implementation settings) can exacerbate existing sleep health disparities [41]. Lack of attention to these important aspects of intervention also contributes to the well-established ~ 17-year gap in translating research to practice [42, 43]. Over the last several decades, researchers have increasingly applied community-engaged research and implementation science approaches to both address research-to-practice gaps and reduce health disparities. Following an introduction to these approaches, we summarize recent examples of pediatric sleep intervention research using community-engaged and/or implementation science principles. We also identify key areas for future research.

### Community-engaged Research (CEnR)

Community-engaged research (CEnR) can address gaps in pediatric sleep care through researcher-community partnerships that integrate the community’s unique expertise, perspectives, lived experience, and local knowledge into research [44, 45]. The Centers for Disease Control and Prevention defines community engagement as an approach that “…builds sustainable relationships through trust and collaboration, strengthening community well-being. The process should be enduring, equitable, and culturally sensitive to all participants, with a shared goal of addressing the concerns of the community” (p. 11) [44]. CEnR can positively impact multiple research areas, including: the relevance of research questions; research design, methods, and ethics; community benefits; and the use and dissemination of research findings [46]. A review of 126 studies funded by the Patient Centered Outcomes Research Institute (PCORI) found that CEnR benefits feasibility, acceptability, rigor, and relevance [47], all of which are important indicators of intervention uptake and sustainability [48].

An important aspect of this work is defining the “community” in question, which may vary considerably depending on the research topic [44]. Communities are not homogeneous, and researchers should consider subgroup perspectives within an identified community [44]. An outpatient mental health clinic, for example, may consist of multiple “communities,” such as clinicians, staff members, and patients/families, with varying perceptions within and across these groups. The extent of community engagement also varies across studies, with scholars conceptualizing CEnR as a continuum ranging from minimal community involvement to community-led projects [45, 49]. Several CEnR continua exist, similarly reflecting increasing researcher-community communication, trust, mutual benefits, and shared power and decision-making with increasing levels of community involvement [45, 49, 50]. Table 1 shows one such continua [49] with added examples of pediatric sleep intervention research at each level. There is some inconsistency in the labels and definitions of community engagements levels in the broader literature [44, 45]. Additionally, the same partnership activity could reflect different levels of engagement. For instance, a community advisory board (CAB) could be consulted once or twice by the research team to *inform* the research procedures or intervention. Reflecting increased engagement, a CAB could *collaborate* with the research team throughout the project period, contributing to all aspects of the research, from generating the scientific question to disseminating findings.

**Table 1:** Examples of community-engaged research activities across a continuum of engagement (Ubri et al. [49])

Level of engagement	Description	Examples
Community Informed	*Community as advisor*: Community members provide information or feedback to guide the research, without a named role on the research team; the community may or may not be aware of the research; communication is mostly unidirectional	• Sleep researcher presents toddler sleep intervention ideas and/or proposed research procedures to a parenting group on sleep training at a pediatrician’s office and asks for feedback; the research team uses this information to refine the research ideas and/or procedures • Caregivers reporting a toddler sleep problem, pediatricians, and/or office staff participate in qualitative research on toddler sleep interventions; the research team integrates their perspectives into intervention content, delivery, and/or research procedures
Community Involved	*Community as collaborator*: Community members are actively involved in some aspect of the research, with a named role on the research team; communication is bidirectional	• Caregivers reporting a toddler sleep problem, pediatricians, and/or office staff are named members of the research team (e.g., as advisory board members) and collaborate with the research team to co-design intervention content, materials, delivery methods, and/or research procedures; pediatricians and office staff assist with research study recruitment
Shared Leadership (Community-Based Participatory Research; Youth-led Participatory Action Research)	*Community as equal partner*: Community members have shared power in project decision-making and ownership, in a named co-leadership role on the research team; communication is bidirectional	• One or more members of the community (e.g., pediatricians at the office, office staff members, caregivers of a toddler with a sleep problem, members of the pediatrician office’s parenting group) co-develop and co-lead the project with a researcher; the community co-owns project results and products
Community Led	*Community as leader*: Community members define the research priorities and questions, lead the research, and engage researchers to support the research	• Pediatrician’s office and members of office’s parenting group aim to deliver and test the effectiveness of a toddler sleep intervention in their setting and seek support of researcher to assist in the evaluation; project results and products are owned by the pediatrician’s office and members of the parenting group

*Community-Based Participatory Research* (CBPR) [51–53] reflects more in-depth, sustained engagement, with a community member or organization co-leading the research. Importantly, CBPR was created in part to redress historical abuse [49] and inequities in research conduct that impacted racial and ethnic minoritized families and/or those marginalized due to lower socioeconomic status [52]. *Youth-led Participatory Action Research* (YPAR) is a related, in-depth CEnR approach in which young people co-develop and co-lead research [54]. Ideally, CEnR approaches yield more equitable health outcomes while also building local capacity and empowering communities to engage in and/or lead future research and advocacy efforts [45, 54]. For example, partnering with community mental health centers to train practicing clinicians in an evidence-based sleep intervention can build organizational capacity to sustain this clinical service over time [55]. To this end, Key et al. highlight equity indicators to consider throughout the partnership, including power and decision-making, respect, mutual benefits, ownership and responsibility of research products, and resource sharing [45]. Especially in the context of health equity research, researcher reflexivity, or ongoing self-reflections about one’s social identities, power, and privilege in relation to the research, is another important aspect of this work [56–58]. Toolkits and training resources are available to help both researchers and community members conduct meaningful CEnR. In addition to resources in this review [44, 45, 49], other examples include: best practices for presenting to CABs, written by CAB members [59]: the PCORI engagement tool and resource repository [60]; the Collaborative Institutional Training Initiative Program CEnR course [61]; the American Academy of Pediatrics (AAP) best practices toolkit for identifying, engaging, and sustaining family advisors [62]; and the University of California, Berkeley, YPAR resource hub [63].

### Implementation Science

Implementation science research focuses on understanding *how* to translate evidence-based practices—including screening tools, interventions, practice guidelines, and policies—into typical care settings for widespread, routine usage [64, 65]. Although not the focus of this review, dissemination science is closely related and similarly concerns the uptake of evidence-based practices, with a more explicit focus on methods to spread knowledge about the use of these practices [66]. As described below, implementation science principles can be incorporated across translational research phases, from designing a new intervention for future implementation to scaling a practice with established effectiveness [65, 67]. Many implementation science theories, models, and frameworks exist to guide this work [68], including theories to explain organizational change mechanisms, frameworks to identify determinants of implementation effectiveness and to evaluate outcomes, and process models to guide the translation of research into practice. Scholars have also recommended incorporating CEnR when conducting implementation science research [41, 67, 69] to avoid perpetuating health disparities when practices are scaled.

**Fig. 1:**
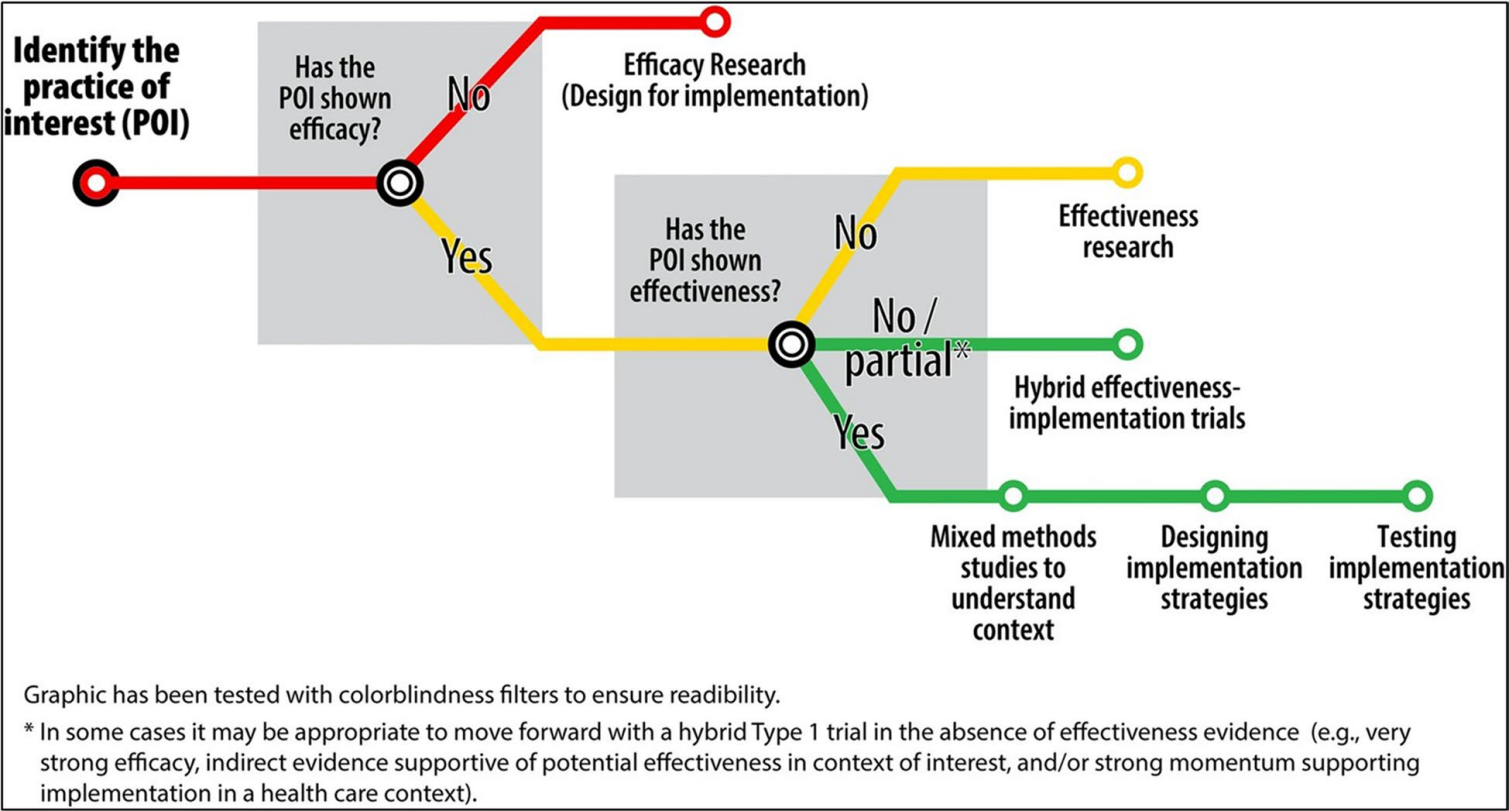
Early to later stage Implementation Science research on the Lane-Fall et al. “Subway line” [65]. Note. This figure from Lane-Fall et al. (2019) [65] is reproduced here under the terms of the Creative Commons CC BY license

Lane-Fall and colleagues provide a helpful “subway line” metaphor (Fig. 1) to illustrate how to navigate from earlier to later stage implementation science research [65]. At the earliest phase, intervention development and efficacy testing can take a “deployment-focused” approach by designing and/or adapting intervention strategies and delivery methods with future scaling in mind [42, 65, 70]. This approach involves considering, for instance, barriers to intervention access and engagement, the training needs of real-world (as opposed to research team-based) interventionists, and whether there are existing care models to inform intervention frequency, duration, and delivery. Implementation outcomes at this phase could include perceptions of intervention *acceptability* and *appropriateness* and whether the intervention is *feasible* and implemented with *fidelity* (i.e., implemented as intended) [48].

For practices with partial or established effectiveness, hybrid effectiveness-implementation trials [71, 72] can be conducted to evaluate patient/family-level impacts (e.g., improvements in child sleep following behavioral intervention) while also evaluating implementation. In addition to examining intervention *acceptability*, *appropriateness*, *feasibility*, and *fidelity*, hybrid trials might examine *adoption* or *uptake* in routine practice, the *costs* of implementation, and whether the intervention can be *sustained* over time (see Proctor et al.) [48]. Hybrid trials are classified into three types based on their relative emphasis on effectiveness versus implementation outcomes [71, 72]. In hybrid type 1 trials, the primary aims are effectiveness-related, with secondary or exploratory implementation aims [72]. Hybrid type 2 trials may have co-primary intervention effectiveness and implementation aims, whereas hybrid type 3 trials focus primarily on implementation outcomes, or the performance of the implementation strategy used [71, 72]. See Curran et al. for questions to consider when selecting a hybrid design [72].

Hybrid type 3 trials and other later stage implementation studies involve developing, selecting, and/or testing implementation strategies, which are distinct from interventions themselves. Implementation strategies are the methods and techniques used to implement an intervention or practice of interest. Whereas earlier stage studies might examine the acceptability and feasibility of *the intervention*, later stage work might also focus on whether *the implementation strategy* used to deploy the intervention is acceptable and feasible. The Expert Recommendations for Implementing Change project provided a refined list of 73 implementation strategies [73]. Strategies range from less intensive approaches, such as developing educational materials (e.g., intervention manuals), to more intensive efforts, such as audit and feedback (e.g., collecting fidelity data and providing reports and feedback to interventionists during a specific period of time) [73]. Strategies can also be single or multi-component. See Powell et al. for additional guidance on methods to select and tailor implementation strategies to improve their fit with the clinical setting [74]. As with CEnR, there are a number of high-quality, freely available resources to learn more about implementation science, which are listed in the National Institutes of Health’s toolkit for implementation researchers [75].

## Recent Examples from Pediatric Sleep Intervention Research

In this section and in Table 2 we identify and describe recent pediatric sleep research examples incorporating CEnR and/or implementation science principles. Examples are organized in text from earlier to later phases of intervention research. This is not an exhaustive list of recent research, and we purposefully include work representing the continuum of CEnR, including studies that sought feedback from participants representing intervention end recipients (e.g., youth, caregivers) and end users (e.g., clinicians) to inform intervention design or refinement. We also include studies that did not explicitly incorporate implementation science principles but examine relevant outcomes (e.g., feasibility, fidelity, acceptability), many of which overlap with those recommended for pilot studies [76, 77].

**Table 2:** Examples of recent research incorporating community-engagement and/or implementation science methods

Practice of interest	Citation(s)	Nature of community engagement	Implementation science principles and/or outcomes
Amsterdam Healthy Sleep Project	Belmon, Busch, et al., [78] Belmon, Brasser, et al., [79] Belmon et al., [80]	• Children ages 9–12 years and caregivers of children ages 4–12 years were research participants in a concept mapping study to identify determinants of poor sleep health in children • Clinicians and sleep experts caring for children ages 4–12 years were research participants in a concept mapping study to identify determinants of poor sleep health in children • Academic institutions and public health services partnered to co-create a sleep health promotion intervention, incorporating concept mapping study results and feedback from planning groups (advisory panel of content experts; community caregiver advisory panel; advisory panel of caregivers working at the public health service)	• *Intervention Mapping [81]: First 4 steps included in Belmon et al. (2022), (1) Logic model of the problem; (2) logic model of change; (3) program design; (4) program production [steps (5) implementation plan and (6) program evaluation plan were not discussed]
ABCs of Sleeping Tool	Howlett et al., [82]	• Caregivers of children with sleep problems and clinicians provided smartphone app feedback as research participants, which the research team used to modify the app	• Outcomes: Intervention acceptability (satisfaction), accessibility, usability
	Jemcov, Keys, & Corkum, [83]	• Caregivers of children with sleep problems provided app feedback as research participants, which the research team used to modify the app	• Outcomes: Intervention acceptability, feasibility, fidelity (use of app)
	Jemcov et al., [84]		• Outcomes: Feasibility of study procedures
BNBD-NDD	Ali et al., [40]	• Sleep experts participated in a Delphi study to generate consensus about key components of online sleep interventions for youth with NDDs	
	Tan-MacNeil, Smith, et al. [85] Tan-MacNeil, Smith, & Corkum, [86]	• Caregivers of children with NDDs and clinicians participated in qualitative research to identify sleep treatment barriers and facilitators as well as the acceptability, pros, and cons of an online intervention for insomnia, which the research team used to inform BNBD-NDD development	• Outcomes: Intervention acceptability
	Jemcov et al., [87] Tan-McNeil et al., [88]	• Caregivers of children with NDDs and insomnia who participated an open pilot of BNBD for typically developing children qualitatively identified barriers and facilitators and rated intervention acceptability, which the research team used to inform BNBD-NDD development	• Authors noted that qualitative implementation barriers and facilitators aligned with the Theoretical Domains Framework [89] for implementation research • Outcomes: Intervention acceptability, feasibility, usability
Charge Your Brainzzz	Heemskerk et al., [90] Heemskerk, van Stralen, et al., [91] Heemskerk, Busch, et al., [92]	• Adolescents, caregivers, and professionals in educational and public health settings participated in a study to identify systems dynamics underlying adolescent sleep health • Adolescents, caregivers, and professionals participated in co-design sessions to identify intervention strategies and possible implementers • Healthy School Advisors, school staff, youth health professionals participated in qualitative research to inform the program components and sleep-friendly school policies; adolescents participated in co-design sessions to finalize intervention components	• *Intervention Mapping [81]: Steps 1 through 4 included across Heemskerk et al. studies [90–92]
DOZE	Carmona et al., [93]	• App development included pilot testing sessions with AYAs, feedback sessions with clinicians, consultation sessions in which AYAs co-designed app elements, and prototype testing with AYAs to finalize the app prior to the open pilot	• Outcomes: Intervention acceptability, feasibility
	Lau & Carney, [94]		• Outcomes: Intervention acceptability, feasibility (engagement)
Safe Sleep Instructor Program+	Ahlers-Schmidt et al., [95] Ahlers-Schmidt et al., [96]	• University-state organization partnership to develop and pilot test a training program that included two evidence-based safe sleep interventions • The state organization provided interventionist training and de-identified study data to the research team	• RE-AIM evaluation framework [97]: Implementation strategy reach, effectiveness, adoption, implementation, maintenance
SIESTA	Koinis-Mitchell et al., [37]	• Latine youth and their caregivers participated in qualitative interviews and an open pilot study, which the research team used to adapt and refine the intervention; school staff consulted with the research team about intervention implementation	• Outcomes: Feasibility of study procedures (open pilot), intervention acceptability, fidelity (RCT)
Sleeping Healthy, Living Healthy	Garbers et al., [98]	• Adolescents and school health center staff participated in a needs assessment and qualitative research to guide intervention development and implementation • Adolescent advisors were research team members and co-designed the intervention and tools for evaluation	
	Bruzzese et al., [99]		• Outcomes: Intervention acceptability, feasibility, fidelity
Sleep Health Intervention for Adolescents (unnamed)	Vandendriessche et al., [100]	• Adolescents from 3 schools were research team members organized into 3 action teams that co-designed the intervention and evaluation plan through a youth participatory action research framework; caregivers also participated in a modified participatory approach to develop caregiver intervention components	• *Intervention Mapping [81]
Sleep Promotion Program	Levenson et al., [101]	• Young people, caregivers, and clinicians participated in qualitative research to guide intervention development and implementation	• CFIR determinants framework [102, 103]: Intervention and implementation barriers, facilitators, and potential adaptations
	Levenson et al., [104]	• Adolescent medicine clinicians were research team members contributing to implementation	• Deployment-focused [42, 70]: Designed as billable service in primary care • Outcomes: Intervention feasibility, acceptability
Sleep Psychology Workshop: Better Sleep for Better Mental Health+	Meaklim et al., [105] Meaklim et al., [106]	• Clinicians in training provided qualitative feedback and acceptability ratings of the workshop	• Outcomes: Implementation strategy acceptability and efficacy • RE-AIM evaluation framework [97]: Implementation strategy reach, effectiveness, adoption, implementation, maintenance
Sleep screener for primary care	Williamson et al., [6]	• Practicing primary care clinicians were research team members and co-designed the sleep screening tool and educational resoufcollaborated as research team members onrces	• RE-AIM evaluation framework [97]: Intervention reach, effectiveness, adoption, implementation, maintenance
SLEEPSMART	Zhai et al., [39]	• Youth with juvenile idiopathic arthritis and caregivers co-designed intervention with the research team	• Outcomes: Intervention acceptability, feasibility
Sleep Well!	Williamson et al., [38]	• Caregivers of children with sleep problems and clinicians participated in qualitative research to guide intervention development and implementation	• CFIR determinants framework [102, 103]: Intervention and implementation barriers, facilitators, and potential adaptations
	Williamson et al.,[107] Williamson et al., [50]	• Clinician advisory board consulted on initial adaptations; family advisory board collaborated as research team members on initial and iterative intervention adaptation, co-design of materials, research procedures for an open pilot and RCT, and interpretation and dissemination of results	• Deployment-focused [42, 70]: Designed as billable service in primary care • Acceptability, cultural humility, feasibility

+Implementation strategy. *Intervention Mapping is not an implementation science theory, model, or framework, but is included because implementation planning is one of the mapping steps, and a similar 6-step Implementation Mapping model is used to develop and plan implementation strategies [108]. *App* = application, *AYAs* = adolescents and young adults, *BNBD-NDD* = Better Nights, Better Days for youth with neurodevelopmental disorders, *DOZE *= Delivering Online Zzz’s with Empirical Support, *NDDs *= neurodevelopmental disorders, *SIETSA *= School Intervention to Enhance Latino Students’ Time Asleep, *RCT *= randomized controlled trial

### Intervention Development, Adaptation, and/or Pilot Evaluations

Several internet-based programs have incorporated youth, caregiver (parents, other family members), and/or clinician feedback during intervention development. To develop the ABCs of Sleeping smartphone application (app) for insomnia, 20 caregivers of 1–12-year-olds with sleep problems and 8 clinicians provided feedback in a usability study [82]. A subsequent open pilot (pretest-posttest) with 23 caregivers found that despite good intervention *acceptability* ratings and child sleep benefits, app use (*fidelity*) was lower than expected, and participants suggested additional app improvements [83]. A pilot randomized controlled trial (RCT) with 28 caregivers evaluated *feasibility* of the study procedures and preliminary app effectiveness [84]. While the app resulted in small sleep and daytime functioning improvements, the 70% recruitment rate and 30% attrition rate suggest that additional modifications could enhance feasibility in a future large-scale trial [84].

Researchers similarly integrated feedback from caregivers and clinicians to adapt the Better Nights, Better Days (BNBD) online insomnia intervention for children with neurodevelopmental disorders (NDDs). Sleep experts identified key program components in a Delphi study [40], while caregivers of children with NDDs and clinicians participated in qualitative research on intervention barriers, facilitators, and *acceptability* [85, 86]. To further adapt BNBD-NDD, researchers drew upon qualitative and quantitative feedback about intervention *acceptability*,* feasibility*, and *usability* from caregivers of children with NDDs who tested the non-adapted BNBD program in an open pilot [87, 88]. The Delivering Online Zzz’s with Empirical Support (DOZE) app for transdiagnostic sleep problems in adolescents and young adults (AYAs) also included end users during development. AYAs and clinicians participated in an iterative co-design process to develop the app [93]. DOZE demonstrated adolescent-rated *acceptability* and *feasibility* (app engagement) in open pilot studies with nonclinical [93] and school-based samples [94]. Recently, SLEEPSMART, a web-based shared-management intervention for youth with juvenile idiopathic arthritis (JIA) and their caregivers, was co-designed in multiple sessions with these ends users. The intervention showed strong *feasibility*, *acceptability*, and improved sleep and self-efficacy in a pilot RCT [39].

In an example of community-engaged sleep intervention adaptation, Koinis-Mitchell et al. [37] conducted a multi-phase, multi-method project to develop a culturally and contextually-tailored school-based sleep health promotion program for Latine middle schoolers in Rhode Island and Puerto Rico. The School Intervention to Enhance Latino Students’ Time Asleep (SIESTA) was adapted from another efficacious adolescent sleep hygiene intervention [109] using Barerra’s 5-stage model [110] for cultural adaptations of behavioral health interventions. These stages included gathering initial in-depth qualitative data from representative end-recipients (Latine adolescents and caregivers), which guided adaptation. The research team also consulted with school staff to plan for school-based pilot testing. SIETSA was further refined through an open pilot trial (*N* = 5) and an RCT (*N* = 34) with Latine adolescents and caregivers. In the RCT, SIESTA showed strong *acceptability* and *fidelity* and improved youth sleep and behavior compared to controls [37].

Some studies have described engaging community members as part of CABs or in other named research team roles. The Amsterdam Healthy Sleep Project [80] brought together universities and public health sectors to develop a child sleep health program using Intervention Mapping (IM) [111] and the Health in All Policies (HiAP) perspective [112], two approaches that emphasize community engagement and sustainable program implementation. The project blueprint for the first 4 of 6 IM steps describes the process of creating multiple CABs representing different stakeholder groups (see Table 2) and conducting a needs assessment with qualitative input from caregivers and professionals. Also in Amsterdam, researchers applied the first 4 IM steps to develop the multi-component Charge Your Brainzzz intervention, incorporating co-design sessions and qualitative feedback from adolescents, caregivers, teachers, school staff, and school-based Healthy Sleep Advisors (interventionists) [90–92].

In the Netherlands, Vandendriessche and colleagues integrated IM and YPAR to co-develop and plan a school-based healthy sleep intervention with 13−15-year-olds as research team members, organized in school-based action teams (*N* = 3–12 participants per team) [100]. The researchers also examined qualitative data from participatory co-design sessions, which highlighted adolescents’ feelings of empowerment and ownership during the partnership process [113]. The quasi-experimental intervention evaluation showed some benefits to adolescent sleep [114]. In another study, researchers partnered with adolescents living in lower-SES New York City neighborhoods to develop Sleeping Healthy, Living Healthy (SHLH) [98, 99], which combines behavioral sleep strategies with mind-body integrative health techniques. Following a needs assessment with adolescents in school-based health centers, two youth joined the research team as advisors, contributing to the intervention development and evaluation plan [98]. Qualitative feedback from adolescents and school staff and school-based pilot testing guided intervention refinement. The pilot RCT (*N* = 61) [99], which explicitly referenced implementation science principles as informing outcomes selection, showed strong adolescent-rated intervention *acceptability*, evidence of *feasibility* (attendance), *fidelity* (ratings of interventionist recordings), and sleep improvements.

Other studies have incorporated specific implementation science frameworks into CEnR intervention design and evaluation. To develop a single-session Sleep Promotion Program (SPP) for adolescent insufficient sleep, Levenson et al. [101] conducted qualitative interviews with young people, caregivers, and clinicians, using the Consolidated Framework for Implementation Research (CFIR) [102, 103] to understand intervention and implementation barriers, facilitators, and potential adaptations [101]. Findings informed the intervention strategies and delivery methods, which were designed for future primary care implementation (i.e., a deployment-focused approach [42, 70]). A RCT of SPP with 44 adolescents showed strong *feasibility* (8.5% attrition), adolescent-rated *acceptability*, improved self-reported sleep [104] and actigraphy-derived regularity in weekend-weekday sleep onset timing [115]. CFIR-informed qualitative interviews and a deployment-focused approach [42, 70] were also used to develop and adapt Sleep Well!, an early childhood intervention for insomnia and insufficient sleep. Qualitative feedback from caregivers of young children with sleep problems and primary care clinicians informed the design of Sleep Well! for implementation as a billable service in primary care and with families experiencing sleep health disparities [50]. A family advisory board representative of families seen at the intervention sites partnered with the research team to co-design intervention materials and delivery methods, and contributed to all aspects of the open pilot [107] and RCT [50, 116], including interpretation and dissemination of findings. Sleep Well! has shown strong *acceptability*, cultural humility, *feasibility*, and improved child sleep problems in the open pilot (*N* = 15 caregiver-child dyads) [107] and RCT (*N* = 97 dyads) [50, 116].

### Intervention Effectiveness and/or Implementation Strategy Evaluations

Very few studies have used CEnR and/or implementation science principles to examine the effectiveness of sleep interventions and implementation strategies. One study [6] used the Reach, Effectiveness, Adoption, Implementation, and Maintenance (RE-AIM) [97] framework to evaluate a sleep screening tool (the practice for interest) for primary care. The EHR-integrated tool was developed with practicing primary care clinician collaborators and designed to align with AAP [117–119] and American Academy of Sleep Medicine [120, 121] practice guidelines for identifying and addressing pediatric sleep problems. When implemented in a 31-practice network, 204,872 of 229,489 patients seen completed the sleep screener (*reach*), with high (≥80%) *adoption* in 89.5% of all well visits. Compared to a period without sleep screening, primary care clinicians were significantly more likely to render a sleep diagnosis and refer children to sleep-related care (*effectiveness*) when using the screener. Few screener adaptations were needed during *implementation*. Adoption remained high (≥80%), at 92.5% during *maintenance*.

In one of few studies to evaluate an implementation strategy, Ahlers-Schmidt and colleagues [96] reported on the impact of the Safe Sleep Instructor (SSI) program for enhancing dissemination of AAP Safe Sleep Recommendations in the state of Kansas. New SSIs received standardized training and certification at a 2-day conference (the implementation strategy). The training was co-developed and pilot-tested through a university and state network partnership [95]. It included two safe sleep education interventions, a Safe Sleep Community Baby Shower [122], developed in a partnership with the Wichita Black Nurses Association, and a Safe Sleep Crib Clinic [123]. Using RE-AIM [97], the study found that the training *reached* 49 individuals, with 44 passing the training post-test at the *a priori* threshold of ≥90% (*effectiveness*) [96]. Since certification, 49% conducted professional trainings and 27% conducted parent/caregiver trainings on safe infant sleep (*adoption*). Regarding *implementation*, individuals trained by a certified SSI (*n* = 903) reported statistically significant increases in relevant knowledge. Lastly, 92% of SSIs recertified one year later (*maintenance*).

While not strictly pediatrics-focused, Meaklim and colleagues developed [105] and evaluated [106] a behavioral sleep education training workshop (the implementation strategy) designed for integration in graduate psychology programs to address gaps in evidence-based insomnia management. The Sleep Psychology Workshop: Better Sleep for Better Mental Health, is a 6-hour online workshop that includes foundational information about sleep, circadian rhythms, and cognitive-behavioral therapy for insomnia (CBT-I). *Acceptability* and initial efficacy were tested in an open pilot with psychology trainees, who also provided qualitative feedback [105]. A state-wide non-randomized waitlist control evaluation [106] using RE-AIM [97] found that the workshop *reached* 313 psychology graduate students. Of the ten graduate programs in the region, seven had students that participated in the workshop (*adoption*). The workshop was perceived as good-to-excellent by 96% of students (*implementation*) and was used in practice by 83% of responding students at 12-month follow-up (*maintenance*). Student participants demonstrated significantly improved sleep knowledge and greater preparedness to conduct sleep care compared to waitlist control (*effectiveness*) [106].

## Summary

This review of recent pediatric sleep intervention research conducted using CEnR and/or implementation science principles demonstrates continued growth in the field and exciting directions for future research. Most studies (Table 2) incorporated CEnR methods during development, adaptation, and/or pilot testing, typically seeking feedback from youth, caregivers, clinicians, and other community members (e.g., school staff, policy makers) [37, 80, 82] that represented the end-recipients and/or end-users for whom the practice of interest (intervention) was designed. Many studies described “co-design” of intervention strategies and/or delivery methods with youth, caregivers, and/or clinicians to incorporate lived experiences and perspectives. However, co-design sessions often occurred with community members as research participants, with fewer studies including community members in named research team roles. Additionally, few studies described community member participation in the interpretation and dissemination of findings. Most studies had small sample sizes, which are appropriate for pilot, qualitative, and user-centered design work, but may limit generalizability. Overall, there is a need for additional, large-scale evaluations of the pediatric sleep interventions developed or piloted using CEnR and/or implementation science principles.

In some cases, the extent of community engagement was difficult to ascertain, in part due to limited discussion of the engagement process, including power dynamics and shared decision-making, researcher-community member communication patterns, and researcher reflexivity [50, 57, 58]. Attention to these equity indicators is particularly important in research with populations experiencing health disparities, to avoid perpetuating historical abuses and harm [45, 52]. As observed by others [54, 124], terminology describing participatory research methods is often used inconsistently. In addition, some studies that sought end user or recipient feedback or included co-design activities referenced applying user-centered design [125, 126], which overlaps with some aspects of CEnR, suggesting some work may not have intentionally incorporated CEnR principles. In future work, clearly describing the extent and process of community engagement in relation to established CEnR continua will help to increase transparency and elucidate the distribution of power and equity across researchers and community members [45, 54]. Accounts of community partners’ perceptions about their engagement in the participatory process were rare [50, 113], and future research should incorporate both qualitative and quantitative approaches to evaluate the engagement process [57].

Although we did not identify any sleep intervention work that has been led by community members or conducted with a community member as a co-leader, some work is underway. The Let’s Yarn About Sleep project protocol [127] describes plans for partnering with Aboriginal and Torres Strait Islander community members (“First Nations Australians”) to develop and evaluate an adolescent sleep health program tailored for First Nations youth and implemented by Aboriginal Youth Workers, to build sustained community capacity for sleep health promotion. Another CBPR project led by a PI with lived experience involves developing and testing culturally tailored sleep intervention for children and families living on the Blackfeet Indian Reservation (K01HL146993; PI: Grant). The Native American Youth Sleep Health and Wellness project, which partnered with American Indian/Alaska Native (AI/NA) community members and a community organization to conduct a longitudinal study, recently generated qualitative data with implications for culturally-tailored sleep interventions for AI/AN youth [128]. More examples of in-depth, sustained CEnR are needed, ideally with members of the end-user or recipient community as co-leaders, or with projects initiated and led by community members and/or organizations.

Very few studies used established implementation science theories, models, or frameworks, and even fewer focused on developing and testing implementation strategies. Most evaluations with implementation outcomes focused on acceptability, feasibility, and fidelity, which overlap with recommended pilot trial outcomes [76, 77]. A recent scoping review of 22 digital sleep interventions for young children [129] also found that implementation outcomes were limited to acceptability, feasibility, and fidelity. Future research should focus on testing sleep interventions with at least partial effectiveness in hybrid trials. Depending on the implementation context, defining *a priori* benchmarks for implementation outcomes (e.g., adoption rate of ≥80%) when possible is also recommended. Several studies used Intervention Mapping to plan intervention evaluations, which could be complemented by the use of Implementation Mapping to design and test implementation strategies [108]. Recent research testing [55] and disseminating [130, 131] implementation strategies for adult sleep interventions are available to guide future pediatric work.

## Conclusions

Incorporating CEnR and implementation science into pediatric sleep intervention research can be challenging. Although implementation science can help speed the translation of sleep science to clinical populations, incorporating CEnR for intervention development and testing is resource- and time-intensive, often with iterative and/or multi-phase procedures. This review identified the need to both invest time in community partnerships in pediatric sleep intervention research and to move the field toward designing and testing implementation strategies. We encourage researchers to balance and integrate these methods to effectively improve pediatric sleep health and address the many gaps in care.

##  Key References

- Ahlers-Schmidt CR, Schunn C, Hervey AM, Torres M, Kuhlmann S, Kuhlmann Z. Using the Reach, Effectiveness, Adoption, Implementation, and Maintenance framework to evaluate a state-wide safe infant sleep education program for continuous improvement. Front Public Health. 2025;13:1540451. doi:10.3389/fpubh.2025.1540451 PMC12380785.
  - **○ **Example of a study that used the RE-AIM framework to evaluate implementation outcomes of a state-wide implementation strategy for safe infant sleep education training.
- Bruzzese JM, Gold MA, Maier MC, et al. Preliminary outcomes of healthy sleep practices and mind-body integrative health intervention among urban youth: Feasibility, acceptability, and initial impact. Sleep Health. 2025;11(6):900–907. doi:10.1016/j.sleh.2025.07.012.
  - **○ **Example of a study that evaluated intervention acceptability, fidelity, and feasibility outcomes of an adolescent sleep health intervention that was co-developed with youth as research team members and with input from key end users and recipients.
- Centers for Disease Control and Prevention & Agency for Toxic Substances and Disease Registry. Principles of community engagement. Third ed. U.S. Department of Health and Human Services; 2025.
  - **○ **Describes and defines community engagement, including key principles and rationale for using this approach, with updates on the latest science included in this third edition.
- Fatima Y, Von Senden R, Bucks RS, et al. A co-designed program for better sleep in Australian First Nations adolescents: protocol for the Let’s Yarn About Sleep adolescent sleep health program. Sleep Adv. Apr 2025;6(2):zpaf012. doi:10.1093/sleepadvances/zpaf012 PMC11983278.
  - **○ **A protocol paper that provides an excellent model for future research by describing a CBPR project co-led by Australian First Nations individuals and a community organization with end goals of community capacity-building and empowerment.
- Harvey AG, Agnew ER, Esteva Hache R, et al. A randomized trial of adapted versus standard versions the transdiagnostic intervention for sleep and circadian dysfunction (TSC) implemented via facilitation and delivered by community mental health providers using train-the-trainer. Implement Sci. Nov 29 2025;21(1):5. doi:10.1186/s13012-025-01467-y PMC12801857.
  - **○ **Example of a large-scale hybrid type 2 effectiveness-implementation trial of an adult sleep intervention, which can be used to guide pediatric sleep intervention research.
- Mitchell MJ, Riley C, Crosby LE. Partnering with Families and Communities to Improve Child Health and Health Equity. Pediatric Clinics. 2023;70(4):683–693.
  - **○ **Describes models, best practices, and guiding principles for community-engaged pediatric intervention research, with case examples and tools for assessing community engagement.
- Vandendriessche A, Verloigne M, Dhondt K, Altenburg TM, Demeester B, Deforche B. Your project or our project? Evaluating the participatory development of an adolescent healthy sleep intervention. BMC Public Health. 2026; 26:28. doi: 10.1186/s12889-025-25667-9.
  - **○ **Example of research examining youth perspectives about the engagement process and their role on the research team in a project that took a youth participatory action research approach to adolescent sleep intervention development.

## Data Availability

No datasets were generated or analysed during the current study.
